# MOG-IgG in NMO and related disorders: a multicenter study of 50 patients. Part 4: Afferent visual system damage after optic neuritis in MOG-IgG-seropositive versus AQP4-IgG-seropositive patients

**DOI:** 10.1186/s12974-016-0720-6

**Published:** 2016-11-01

**Authors:** Florence Pache, Hanna Zimmermann, Janine Mikolajczak, Sophie Schumacher, Anna Lacheta, Frederike C. Oertel, Judith Bellmann-Strobl, Sven Jarius, Brigitte Wildemann, Markus Reindl, Amy Waldman, Kerstin Soelberg, Nasrin Asgari, Marius Ringelstein, Orhan Aktas, Nikolai Gross, Mathias Buttmann, Thomas Ach, Klemens Ruprecht, Friedemann Paul, Alexander U. Brandt, in cooperation with the Neuromyelitis Optica Study Group (NEMOS)

**Affiliations:** 1 NeuroCure Clinical Research Center (NCRC), Charité – Universitätsmedizin Berlin, Charitéplatz 1, 10117 Berlin, Germany; 2 Department of Neurology, Charité – Universitätsmedizin Berlin, Berlin, Germany; 3 Molecular Neuroimmunology Group, Department of Neurology, University of Heidelberg, Heidelberg, Germany; 4 Clinical Department of Neurology, Medical University of Innsbruck, Innsbruck, Austria; 5 Division of Neurology, Children’s Hospital of Philadelphia, Pennsylvania, USA; 6 Department of Neurology, Vejle Hospital, Vejle, Denmark; 7 Department of Neurobiology, Institute of Molecular Medicine, University of Southern Denmark, Odense, Denmark; 8 Department of Neurology, Medical Faculty, Heinrich Heine University, Düsseldorf, Germany; 9 Department of Ophthalmology, Medical Faculty, University of Freiburg, Freiburg, Germany; 10 Department of Neurology, University of Würzburg, Würzburg, Germany; 11 Department of Ophthalmology, University of Würzburg, Würzburg, Germany; 12 Experimental and Clinical Research Center, Max Delbrück Center for Molecular Medicine and Charité – Universitätsmedizin Berlin, Berlin, Germany

**Keywords:** Myelin oligodendrocyte glycoprotein antibodies (MOG-IgG), aquaporin-4 antibodies (AQP4-IgG), NMO-IgG, neuromyelitis optica, Devic syndrome, neuromyelitis optica spectrum disorders (NMOSD), optic neuritis, optical coherence tomography, visual evoked potentials, visual acuity, retinal neuro-axonal damage

## Abstract

**Background:**

Antibodies against myelin oligodendrocyte glycoprotein (MOG-IgG) have been reported in patients with aquaporin-4 antibody (AQP4-IgG)-negative neuromyelitis optica spectrum disorders (NMOSD). The objective of this study was to describe optic neuritis (ON)-induced neuro-axonal damage in the retina of MOG-IgG-positive patients in comparison with AQP4-IgG-positive NMOSD patients.

**Methods:**

Afferent visual system damage following ON was bilaterally assessed in 16 MOG-IgG-positive patients with a history of ON and compared with that in 16 AQP4-IgG-positive NMOSD patients. In addition, 16 healthy controls matched for age, sex, and disease duration were analyzed. Study data included ON history, retinal optical coherence tomography, visual acuity, and visual evoked potentials.

**Results:**

Eight MOG-IgG-positive patients had a previous diagnosis of AQP4-IgG-negative NMOSD with ON and myelitis, and eight of (mainly recurrent) ON. Twenty-nine of the 32 eyes of the MOG-IgG-positive patients had been affected by at least one episode of ON. Peripapillary retinal nerve fiber layer thickness (pRNFL) and ganglion cell and inner plexiform layer volume (GCIP) were significantly reduced in ON eyes of MOG-IgG-positive patients (pRNFL = 59 ± 23 μm; GCIP = 1.50 ± 0.34 mm^3^) compared with healthy controls (pRNFL = 99 ± 6 μm, *p* < 0.001; GCIP = 1.97 ± 0.11 mm^3^, *p* < 0.001). Visual acuity was impaired in eyes after ON in MOG-IgG-positive patients (0.35 ± 0.88 logMAR). There were no significant differences in any structural or functional visual parameters between MOG-IgG-positive and AQP4-IgG-positive patients (pRNFL: 59 ± 21 μm; GCIP: 1.41 ± 0.27 mm^3^; Visual acuity = 0.72 ± 1.09 logMAR). Importantly, MOG-IgG-positive patients had a significantly higher annual ON relapse rate than AQP4-IgG-positive patients (median 0.69 vs. 0.29 attacks/year, *p* = 0.004), meaning that on average a single ON episode caused less damage in MOG-IgG-positive than in AQP4-IgG-positive patients. pRNFL and GCIP loss correlated with the number of ON episodes in MOG-IgG-positive patients (*p* < 0.001), but not in AQP4-IgG-positive patients.

**Conclusions:**

Retinal neuro-axonal damage and visual impairment after ON in MOG-IgG-positive patients are as severe as in AQP4-IgG-positive NMOSD patients. In MOG-IgG-positive patients, damage accrual may be driven by higher relapse rates, whereas AQP4-IgG-positive patients showed fewer but more severe episodes of ON. Given the marked damage in some of our MOG-IgG-positive patients, early diagnosis and timely initiation and close monitoring of immunosuppressive therapy are important.

**Electronic supplementary material:**

The online version of this article (doi:10.1186/s12974-016-0720-6) contains supplementary material, which is available to authorized users.

## Background

Myelin oligodendrocyte glycoprotein (MOG) is expressed on the outer surface of oligodendrocytic myelin sheaths, representing approximately 0.05 % of all myelin-constituting proteins [1]. Antibodies against MOG (MOG-IgG) have been detected in a proportion of aquaporin-4 (AQP4)-IgG-seronegative patients with neuromyelitis optica spectrum disorder (NMOSD) phenotype [2–6]. MOG-IgG have further been reported in children with acute and relapsing-remitting inflammatory demyelinating encephalomyelitis as well as in a proportion of adults with inflammatory demyelinating diseases such as optic neuritis (ON) [7–9].

Currently it is debated whether MOG-IgG-associated encephalomyelitis should be classified as an NMOSD subtype or as a separate disease entity [10–12]. MOG-IgG-seropositive patients from NMOSD cohorts can show clinical features of recurrent transverse myelitis and ON, similar to AQP4-IgG-seropositive patients [4]. However, the cellular target of AQP4-IgG is an astrocytic water channel, suggesting a different mechanism of injury from MOG-IgG. This is supported by a recent case study of a MOG-IgG-seropositive patient who showed severe demyelination with no evidence of astrocytopathy [13] and by further brain biopsy case studies [14–16].

ON in NMOSD patients is often severe with marked retinal nerve fiber layer and ganglion cell layer loss, severe visual impairment including blindness, and a high frequency of bilateral events [17, 18]. In around 20 % of affected eyes, macular microcysts are found in the inner nuclear layer as a sign of severe ON-related retinal injury [19, 20]. In comparison, the extent of afferent visual system damage following ON in MOG-IgG-seropositive patients is less well understood.

Some previous studies, employing either structural or clinical assessment of visual function, suggested that MOG-IgG-positive patients have fewer attacks, better recovery from relapses, and less neuro-axonal retinal damage than AQP4-IgG-positive patients [4, 21, 22]. However, it is a potential drawback that observation periods were relatively short and sample sizes low in those studies. Moreover, some included mostly or exclusively Asian patients [4, 22]; this could be relevant in that genetic factors have been proposed to play a role in NMOSD pathogenesis [17]. By contrast, more recent studies by others [23, 24] and us [25] demonstrate that the disease follows a relapsing course in the long run in most MOG-IgG-positive patients.

The objective of this retrospective multicenter study was to investigate visual system damage after ON in a larger cohort of Caucasian patients with MOG-IgG-associated encephalomyelitis and long-term follow-up using a comprehensive assessment of the afferent visual system including structural, functional, and clinical parameters, and to compare it with that in AQP4-IgG-positive NMOSD patients.

## Methods

### Patients

MOG-IgG-seropositive patients with a history of ON and available optical coherence tomography (OCT) data were recruited from a large retrospective study [25, 26]. Sixteen patients (15 female; mean age 44.0 ± 15.2 years) were enrolled from six university hospitals in Europe (Germany: Berlin, Freiburg, Düsseldorf, Heidelberg, Würzburg; Denmark: Vejle). The inclusion criteria were age ≥18 years, a confirmed history of ON (more than 3 months prior to visual assessments), and seropositivity for MOG-IgG. A MOG-antibody serum titer of ≥1: 160 was classified as positive [26]. Clinical and paraclinical data on disease onset, relapse history, expanded disability status scale (EDSS) [27], visual acuity, OCT, magnetic resonance imaging (MRI), and immunotherapy were retrospectively collected.. Annualized relapse rate was calculated as the ratio of number of attacks and years since disease onset, excluding patients with disease duration of less than 1 year. All patients were of Caucasian descent; all MOG-IgG-positive patients tested seronegative for AQP4-IgG, and vice versa (Table 1). Eight (50 %) MOG-IgG-positive patients had a previous diagnosis of—mainly recurrent—ON, and eight (50 %) had been diagnosed with NMOSD based on the clinical symptoms of ON and myelitis before anti-MOG-IgG was tested. AQP4-IgG-positive NMOSD patients [28] (*n* = 16, all female, mean age 43.2 ± 13.9 years) and healthy controls (HC, *n* = 16, 15 female, mean age 43.9 ± 15.4 years) were randomly selected from the research database of the NeuroCure Clinical Research Center (Charité – Universitätsmedizin Berlin, Berlin, Germany), matched for sex and age on cohort basis. Two MOG-IgG-positive patients had co-occurring ophthalmologic conditions in both eyes: one had early-stage dry macular degeneration, and glaucoma was suspected in the other patient. These two patients and their matched AQP4-IgG-positive patients and HC were included in the case descriptions but excluded from statistical analyses of OCT and visual function parameters. Furthermore, only eyes with a previous ON were included in statistical analyses. The local ethics committees approved the study protocol in accordance with the Declaration of Helsinki (1964) in its currently applicable version. All participants provided informed written consent.

**Table 1:** Demographic data

		MOG-IgG	AQP4-IgG	MOG-IgG vs. AQP4-IgG (MWU/Chi^2^)
				*p*
Patients	*N*	16	16	
Age (years)	Mean ± SD	44.0 ± 15.2	43.2 ± 13.9	0.838
Sex (f/m)		15/1	16/0	>0.999
Ophthalmologic comorbidities	*N*	2^a)^ (13 %)	0 (0 %)	
Age at onset (years)	Mean ± SD	37.2 ± 15.1	34.7 ± 14.8	0.669
Time since onset (years)	Mean ± SD	6.9 ± 6.5	8.4 ± 6.8	0.287
ON eyes	*N* (%)	29 (91.6 %)	25 (78.1 %)	
Number of ON episodes	Median (range)	4.5 (1–13)	2 (1-4)	**0.012**
Myelitis prevalence	*N* (%)	8 (50 %)	15 (93.8 %)	**0.018**
ARR	Median (range)	1.25 (0.38–7.14)	0.64 (0.17–1.44)	**0.026**
ON ARR	Median (range)	0.69 (0.17–7.14)	0.29 (0.07–0.96)	**0.004**
EDSS	Median (range)	3.0 (1.0–7.5)	4.0 (1.0–6.5)	0.064

*Abbreviations*: *AQP4-IgG* aquaporin-4 antibody-seropositive NMOSD patients, *ARR* annualized relapse rate, *EDSS* expanded disability status scale, *f* female, *m* male, *MOG-IgG* myelin oligodendrocyte glycoprotein antibody-seropositive patients, *MWU* Wilcoxon-Mann-Whitney *U* test, *ON* optic neuritis, *SD* standard deviation

^a)^Early stage dry macular degeneration in both eyes and suspect for early stage glaucoma, respectively

*p*-values in bold emphasis depict significant values (p < 0.05)

### MOG-IgG and AQP4-IgG assay

MOG-IgG antibodies were detected using a live cell-based assay and a fixed cell-based assay, both employing HEK293 cells transfected with human full-length MOG; mock-transfected cells were used as control substrates (see part 1 for details [26]). AQP4-IgG were detected using a commercially available cell-based assay (EUROIMMUN, Lübeck, Germany) [29, 30].

### Optical coherence tomography

**Fig. 1:**
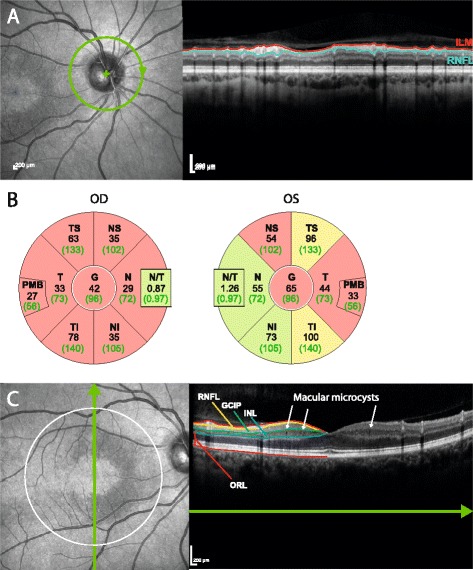
Sample images from patient 1. **a** Sample images from a peripapillary ring scan. On the *left*, a scanning laser ophthalmoscopy image shows scan positioning (in *green*). On the *right*, an OCT scan shows severe peripapillary retinal nerve fiber layer (pRNFL) loss (between the inner limiting membrane [ILM], shown in *red*, and the lower border, in *turquoise*). **b** Ring-scan data in comparison with normative device data from both eyes of this patient. *Black numbers* display the thickness measurements (in μm) of the subject, *green numbers* the average thickness in the age-matched reference group. Sectors are classified in comparison with the reference group: *green*, thickness values within the 5th and 95th percentile range; *yellow*, 1st to 5th percentile range; *red*, below the 1st percentile. *Abbreviations*: *G* global, *NS* nasal-superior, *N* nasal, *NI* nasal-inferior, *TI* temporal-inferior, *T* temporal, *TS* temporal-superior. **c** Macular scan of the same patient. On the *left*, the dark, sickle-shaped area on and around the macula represents tissue with microcysts in the inner nuclear layer (INL). The *white circle* indicates the 6-mm-diameter cylinder in which intraretinal layers are analyzed. The *green line* with *arrow* shows the scanning position of the OCT scan on the right. Here, the defined layers are the RNFL, the ganglion cell and inner plexiform layer (GCIP), then INL and the outer retinal layers (ORL). Macular microcysts can be seen as small *black dots* in the INL

OCT was performed using the Spectralis SD-OCT device (Heidelberg Engineering, Heidelberg, Germany) with the automatic real time function for image averaging. Peripapillary retinal nerve fiber layer thickness (pRNFL) was derived from a standard ring scan around the optic nerve head (12°, 768 or 1536 A-scans, 16≤ART≤100). A macular volume scan (25° × 30°, 61 vertical or horizontal B-scans, 768 A-scans per B-scan, 9≤ART≤15) was acquired for retinal layer analysis. All scans underwent quality control [31] and post-processing by one experienced rater in a standardized manner. Layer segmentation was performed with the device’s software (Eye Explorer 1.9.10.0 with viewing module 6.0.9.0). Automatic segmentation results were carefully checked for errors and corrected if necessary by an experienced rater masked for the diagnosis of the subjects. Combined ganglion cell and inner plexiform layer volume (GCIP), inner nuclear layer volume, and outer retinal layers volume including the outer plexiform and nuclear layer, inner and outer photoreceptor segments, and retinal pigment epithelium, were extracted from a 6-mm-diameter cylinder around the fovea [32]. Furthermore, all scans were examined for macular microcysts [19] and other retinal pathologies. The OCT parameters are visualized in Fig. 1.

### Visual function testing

Visual function testing was performed in MOG-IgG-positive and AQP4-IgG-positive patients at the same visit as OCT, except for one patient (see Additional file 1: Table S1). Visual evoked potentials (VEP) were recorded with checkerboard stimulation (1°) with the device routinely used at the sites. P100 peak latency was included in analysis and considered as abnormal when higher than 112 ms [33] or when no clear signal could be evoked. Habitually corrected visual acuity was tested with letter charts obtained as part of routine clinical care and converted into logMAR units. A visual acuity of 0.2 logMAR and worse was considered abnormal. When no letter could be recognized by the patient, visual acuity was registered with 2.0 logMAR for finger counting and 3.0 logMAR for hand motion recognition [34].

### Data analysis

Statistics were performed in R version 3.1.2 [35] using the packages psych, MASS, geepack and ggplot. Differences in demographics between the cohorts were tested with Pearson chi-square test and non-parametric tests (Mann-Whitney U for two cohorts and Kruskal-Wallis for three cohorts). Comparisons of visual system data between cohorts were performed using generalized estimating equation (GEE) models accounting for intra-subject inter-eye dependencies. GEE results are provided with regression coefficient (B) and standard error (SE). To investigate the extent of damage caused by subsequent ON episodes we employed a linear spline regression model as proposed by Ratchford et al. [36]. Due to the exploratory nature of this study, no correction for multiple comparisons was performed.

## Results

The demographic and clinical features of MOG-IgG-positive patients are presented in Table 1 and case-by-case clinical details are provided in Additional file 1: Table S1. One patient had pediatric onset of the disease, at 6 years of age; her case has been reported in an earlier publication [11]. All other patients had adult onset. All MOG-IgG-positive patients had experienced at least one episode of ON (median 4.5, range 1–13) and, except for one with a short follow-up period (8 months, patient 8), presented with an unequivocally relapsing disease course. Age at onset and disease duration at the time of examination did not differ between MOG-IgG-positive and AQP4-IgG-positive patients (Table 1). Detailed case studies, including therapy, are provided in parts 2 and 3 of this series of articles [25, 37].

### OCT and visual function in MOG-IgG-positive ON

Two eyes from two patients had to be excluded from the analysis owing to acute ON at the time of assessment. Thus, 23 eyes from 14 MOG-IgG-positive patients were analyzed at a median time of 16.4 months (range 3–125 months) since the most recent episode of ON. Detailed afferent visual system parameters of all patients are given in Table 2, and case-by-case descriptions are provided in Additional file 2: Table S2.

**Table 2:** Structural and functional data of MOG-IgG-positive patients’ ON eyes in comparison to AQP4-IgG-positive patients and control data

	MOG-IgG positive ON (*n* = 23 eyes from 14 subjects)	AQP4-IgG positive ON (*n* = 21 eyes from 14 subjects)	HC (*n* = 28 eyes from 14 subjects)	MOG-IgG positive vs. AQP4-IgG positive (GEE)			MOG-IgG positive vs. HC (GEE)		
				B	SE	*p*	B	SE	*p*
Retinal OCT									
Average pRNFL (μm)	59 ± 23	59 ± 21	99 ± 6	−0.6	7.58	0.94	39.0	6.01	**<0.001**
Nasal pRNFL (μm)	44 ± 21	45 ± 24	74 ± 12	0.2	7.85	0.98	28.6	6.01	**<0.001**
Temporal pRNFL (μm)	44 ± 16	40 ± 15	71 ± 10	−3.0	4.51	0.50	27.6	4.26	**<0.001**
GCIP (mm^3^)	1.50 ± 0.34	1.41 ± 0.27	1.97 ± 0.11	−0.10	0.10	0.35	0.47	0.08	**<0.001**
INL (mm^3^)	1.03 ± 0.10	1.01 ± 0.11	0.95 ± 0.04	−0.02	0.04	0.55	−0.07	0.03	**0.009**
ORL (mm^3^)	4.86 ± 0.26	4.93 ± 0.26	4.73 ± 0.21	0.04	0.09	0.70	−0.13	0.09	0.14
Eyes with macular microcysts (*n*)	5 (21.7 %)	4 (19.0 %)		Chi^2^		>0.99			
Visual function									
Visual acuity/logMAR	0.35 ± 0.88	0.72 ± 1.09	-	0.33	0.32	0.30			
Abnormal P100 latency*	12 (57 %)	10 (50 %)	-	Chi^2^		0.88			

OCT and visual function results are not including data from the two patients with early stage dry macular degeneration in both eyes and glaucoma, respectively, and their respective AQP4-IgG-positive controls and healthy controls. Furthermore, two eyes of two MOG-IgG positive patients were excluded due to acute ON at time of examination. Explanations: All data are given as mean ± standard deviation (minimum – maximum), if not declared different

*AQP4-IgG* aquaporin-4 antibody-seropositive NMOSD patients, *GCIP* ganglion cell and inner plexiform layer, *HC* healthy controls, *INL* inner nuclear layer, *ON* eyes with history of optic neuritis, *MOG-IgG* myelin oligodendrocyte glycoprotein antibody-seropositive patients, *ORL* outer retinal layers including layer from outer plexiform layer to Bruch’s membrane, *pRNFL* peripapillary retinal nerve fiber layer

*p*-values in bold emphasis depict significant values (*p* < 0.05)

* VEP data were available for 20 out of 23 ON eyes of MOG-IgG positive patients and 20 out of 21 eyes of AQP4-IgG positive patients

Reduced pRNFL thickness compared with the manufacturer’s normative data was found in 18 of the 23 (78.2 %) ON-affected eyes of the MOG-IgG-positive group (mean 59 ± 23 μm). In addition, two fellow eyes without clinically evident previous ON and with normal VEPs showed reduced RNFL thickness (51 μm and 75 μm, respectively). Five ON eyes (21.7 %) but none of the non-ON eyes had macular microcysts in the inner nuclear layer. Of 20 ON eyes with available VEP data, 12 (60 %) eyes had abnormal P100 latencies—two (10 %) of them despite normal pRNFL—while all four non-ON fellow eyes had normal VEPs. Visual acuity was on average reduced in ON eyes (mean 0.35 ± 0.88 logMAR), with three eyes being legally blind at a visual acuity of 1.0 logMAR and worse. On the other hand, 16 of 23 ON eyes (70 %) preserved visual acuity of 0.1 logMAR or better.

There were no significant differences in OCT and visual function measurements between MOG-IgG-positive patients with a history of both ON and myelitis (*n* = 8) and MOG-IgG-positive patients with a history only of recurrent ON (*n* = 8) (not shown).

### Comparison with HC and AQP4-IgG-positive NMOSD patients

**Fig. 2:**
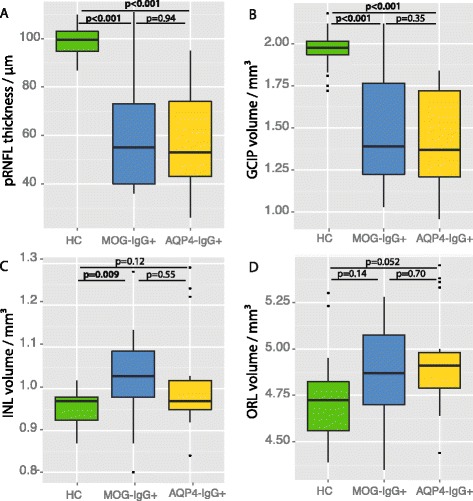
Retinal layer measures of MOG-IgG-positive and AQP4-IgG-positive ON eyes. *Boxplots* for the comparison of retinal layer measures of the eyes in the healthy control group and the ON eyes of MOG-IgG-positive (MOG-IgG+) and AQP4-IgG-positive (AQP4-IgG+) NMOSD patients. (**a**) Peripapillary retinal nerve fiber layer thickness derived from a ring scan (pRNFL); (**b**-**d**) Intraretinal layer volumes quantified in a 6-mm-diameter cylinder around the fovea centralis: (**b**) ganglion cell and inner plexifom layer volume (GCIP); (**c**) inner nuclear layer volume (INL); (**d**) outer retinal layer volume comprising all layers from outer plexiform layer to Bruch’s membrane

We then compared the afferent visual system damage in ON eyes of MOG-IgG-positive patients with age- and sex-matched HC and with ON eyes of AQP4-IgG-positive NMOSD patients (Table 2, Fig. 2). As expected, pRNFL and GCIP were significantly lower than in HC both in the MOG-IgG-positive group (both *p* < 0.001) and in the AQP4-IgG-positive group (both *p* < 0.001). Furthermore, inner nuclear layer volume was significantly greater than HC in the MOG-IgG-positive subgroup (*p* = 0.009), but not in the AQP4-IgG-positive NMOSD subgroup. By contrast, no significant difference was noted between MOG-IgG-positive and AQP4-IgG-positive patients regarding retinal layer measures. Macular microcysts were found in both subgroups in similar prevalence, but differences in microcyst size or extend might have led to a high variability of inner nuclear layer volume values (Table 2). Visual acuity was less impaired in the MOG-IgG-positive subgroup (mean 0.35 ± 0.88 logMAR) than in the AQP4-IgG-positive subgroup (0.72 ± 1.09); however, the difference was not significant (*p* = 0.30).

Of note, the MOG-IgG-positive patients showed a significantly higher annualized relapse rate both for all relapses and—even higher—for ON than the AQP4-IgG-positive patients (*p* = 0.026 and *p* = 0.004, respectively), despite similar disease duration (Table 1).

### Retinal damage and number of ON episodes

In MOG-IgG-positive patients, a higher number of ON episodes was associated with more severe pRNFL and GCIP loss (GEE: pRNFL B = −4.9, SE = 1.40, *p* < 0.001; GCIP B = −0.07, SE = 0.02, *p* < 0.001), but not with changes of the inner nuclear layer or outer retinal layers. By contrast, in AQP4-IgG-positive patients the extent of retinal layer changes did not correlate with the number of ON attacks.

**Fig. 3:**
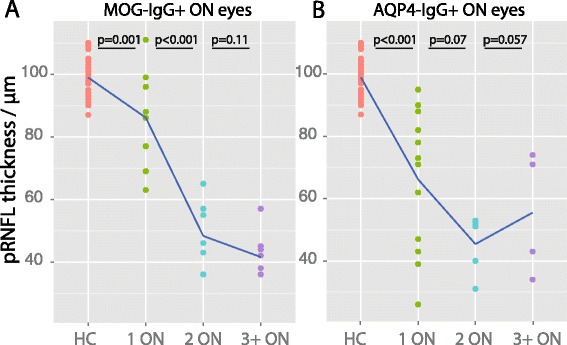
Retinal nerve fiber layer loss as a function of optic neuritis in MOG-IgG-positive and AQP4-IgG-positive patients. Peripapillary retinal nerve fiber layer (pRNFL) loss caused by sequential episodes of optic neuritis (ON), estimated from cross-sectional data, in comparison with eyes without optic neuritis from the healthy control (HC) cohort. (**a**) ON eyes from MOG-IgG-seropositive patients (MOG-IgG+); (**b**) ON eyes from AQP4-IgG-seropositive patients (AQP4-IgG+). *P*-values were computed with linear regressions

In our cross-sectional data, the first ON episode caused a mean pRNFL loss of 12.8 μm (*p* = 0.001) in MOG-IgG-positive patients and 32.8 μm (*p* < 0.001) in AQP4-IgG-positive patients in comparison with HC eyes. In contrast, a second episode of ON caused additional pRNFL loss of 37.8 μm (*p* < 0.001) in MOG-IgG-positive patients and 20.8 μm in AQP4-IgG-positive patients, although that difference was not significant (*p* = 0.07) (Fig. 3). A similar association was found for GCIP volume (data not shown).

## Discussion

This study shows that ON in MOG-IgG-positive patients leads to severe pRNFL and GCIP thinning and visual function impairment, the extent of which is comparable to ON in patients with AQP4-IgG. Moreover, it suggests that the damage accrual may be driven by higher relapse rates in MOG-IgG-positive patients, in contrast to more severe ON-associated damage during a single ON episode in AQP4-IgG-positive patients.

Some earlier studies of MOG-IgG-positive patients, which were characterized by relatively short observation periods, suggested that MOG-IgG-seropositive patients present more often with monophasic disease and have a milder clinical phenotype and better recovery than patients with AQP4-IgG-seropositive NMOSD [4, 5, 38]. By contrast, all but one of our patients showed a relapsing disease course with a high frequency of attacks, protracted ON episodes, and, in some cases, severe visual impairment. In line with our findings, two more recent studies have also demonstrated that MOG-IgG seropositivity is frequently associated with a recurrent disease course in patients with ON [23, 24]. Concerning neuro-axonal damage of the retina, a recent study including 19 MOG-IgG-positive patients reported less retinal nerve fiber and ganglion cell layer damage than in AQP4-IgG-positive patients following ON [22]. As a limitation, however, that study included exclusively monophasic patients. By contrast, in our study we demonstrated that retinal neuro-axonal damage after ON in MOG-IgG-positive patients is at least as severe as in AQP4-IgG-positive NMOSD patients, compared with our own control cohort as well as with previously published AQP4-IgG-positive cohorts [39, 40] when patients with long-term follow-up (mean ~7 years) and, accordingly, relapsing disease course are included in the analysis.

Notably, although average visual function was impaired in relapsing ON of both MOG-IgG-positive and AQP4-IgG-positive patients, some MOG-IgG-positive patients performed comparably well on high-contrast visual acuity testing despite severe neuro-axonal retinal damage: 70 % of ON eyes retained a visual acuity of 0.1 logMAR or better after ON. However, visual acuity was obtained in non-standardized manner as high-contrast letter acuity in clinical routine; thus the reliance on functional testing may underestimate the actual extent of damage to the afferent visual system. The impact of structural damage as demonstrated in the present study should be further investigated with low-contrast letter acuity, color vision testing, visual fields, and quality of life scales.

Our study features strengths and limitations. Among its strengths we count the relatively high number of patients included, given the low prevalence of the disease, the fact that reliable assays for detecting antibodies to full-length human MOG have become available only relatively recently, and the fact that OCT is not yet routinely and generally available. A further potential strength is that our cohort was genetically homogeneous with all patients and controls being of Caucasian origin. As a potential limitation, not all patients were systematically tested for other optic neuropathies, such as Leber’s hereditary optic neuropathy (LHON). While a mitochondrial mutation may have contributed to the marked pRNFL thinning in the female patient with pediatric onset of disease (patient 4 in Additional file 1: Table S1), the time course (approximately 10 years before the contralateral eye demonstrated a mild decrease in visual acuity) is unusual for LHON, a condition which typically affects both eyes within months of each other without a relapsing and remitting course. Finally, data were collected retrospectively in a multicenter approach. As a result, additional data, e.g., the Multiple Sclerosis Function Composite or OCT scans obtained during acute optic neuritis, were not available. Moreover, we were not able to systematically correlate optic nerve MRI [23, 41] and OCT in this study, which would require highly standardized MRI protocols and a prospective study design. However, prospective studies as well as single-center studies in MOG-IgG-positive patients are difficult to perform due to the condition’s rarity and the currently limited access to MOG-IgG testing. Moreover, all patients with available data seen at the various centers were included in the analysis, thereby reducing the risk of referral bias. Nonetheless, the preliminary evidence derived from this retrospective exploratory study needs to be confirmed in further prospective and independent studies.

## Conclusions

In summary, we demonstrate (a) that a substantial proportion of MOG-IgG-seropositive patients develop retinal neuro-axonal damage; (b) that visual impairment and structural damage increase with the number of attacks and thus with disease duration; and, importantly, (c) that the extent of neuro-axonal damage in MOG-IgG-positive patients with ON is not different from that in patients with AQP4-IgG-positive ON in the long-term course of the disease, i.e., when patients with relapsing rather than monophasic ON are taken into account. Given the marked structural and functional damage in some of our ON patients, early diagnosis, timely initiation of immunosuppressive therapy, and close monitoring of treatment efficacy seem paramount. Although no systematic investigations of drugs for relapse prevention in this condition have yet been conducted, retrospective data on treatment responses (see part 2 of this series [25]), as well as available evidence in favor of a pathogenic role of MOG-IgG [16, 30], suggest that—in accordance with treatment recommendations for AQP4-IgG-positive NMOSD [42]—patients with MOG-IgG-positive ON may benefit from high-dose intravenous methylprednisolone treatment and, possibly, plasma exchange for acute attacks as well as from immunosuppression for attack prevention.

## Additional files

Additional file 1: Table S1. Demographic, clinical and serological data. ^a)^ Early stage dry macular degeneration in both eyes; ^b)^ Suspected early stage glaucoma. ^c)^ Visual assessments were performed during acute ON OS. Abbreviations: ON: optic neuritis. VEP P100: visually evoked potential P100 latency. n.e.: not evocable; pRNFL: peripapillary retinal nerve fiber layer thickness. GCIP: combined ganglion cell and inner plexiform layer volume. INL: inner nuclear layer volume. ORL: outer retinal layers volume including layers from outer plexiform layer to Bruch’s membrane. (DOCX 21 kb)
Additional file 2: Table S2. Visual evoked potentials, visual acuity, and OCT results. ^a)^ Protracted relapses were registered as one episode; ^b)^ Early stage dry macular degeneration in both eyes; ^c)^ Suspected early stage glaucoma; ^d)^ Medication other than acute relapse therapy (immunotherapy). Abbreviations: AQP4-IgG = aquaporin-4 antibodies; CRION = chronic relapsing inflammatory optic neuropathy; EDSS = expanded disability status scale; F = female; MOG-IgG = myelin oligodendrocyte glycoprotein antibodies; NMOSD = neuromyelitis optica spectrum disorders; (r)ON = (recurrent) optic neuritis. (DOC 59 kb)

## Abbreviations

AQP4-IgG: aquaporin-4 immunoglobulin G
ARR: annualized relapse rate
EDSS: expanded disability status scale
GCIP: ganglion cell and inner plexiform layer volume
GEE: generalized estimating equation
LHON: Leber’s hereditary optic neuropathy
MOG-IgG: myelin oligodendrocyte glycoprotein antibody-seropositive patients
MWU: Wilcoxon-Mann-Whitney *U* test
OCT: optical coherence tomography
ON: optic neuritis
pRNFL: peripapillary retinal nerve fiber layer thickness
SD: standard deviation
SE: standard error
VEP: visual evoked potentials
