# Supplementary material for: MOG-IgG in NMO and related disorders: a multicenter study of 50 patients. Part 4: Afferent visual system damage after optic neuritis in MOG-IgG-seropositive versus AQP4-IgG-seropositive patients
Source: J Neuroinflammation. 2016 Nov 1;13:282. doi: 10.1186/s12974-016-0720-6 (PMC5088645; doi:10.1186/s12974-016-0720-6)
Supplement: Additional file 1: Table S1. — Demographic, clinical and serological data. a) Early stage dry macular degeneration in both eyes; b) Suspected early stage glaucoma. c) Visual assessments were performed during acute ON OS. Abbreviations: ON: optic neuritis. VEP P100: visually evoked potential P100 latency. n.e.: not evocable; pRNFL: peripapillary retinal nerve fiber layer thickness. GCIP: combined ganglion cell and inner plexiform layer volume. INL: inner nuclear layer volume. ORL: outer retinal layers volume including layers from outer plexiform layer to Bruch’s membrane. (DOCX 21 kb) [file 12974_2016_720_MOESM1_ESM.docx]

| **Patient No.** | 1 | 2 | 3 | 4 | 5 | 6 | 7 | 8 | 9 | 10 | 11 | 12 | 13 | 14 | 15 | 16 |
| --- | --- | --- | --- | --- | --- | --- | --- | --- | --- | --- | --- | --- | --- | --- | --- | --- |
| **Case in part 2 and 3 [25,37]** | Part 2, case 17 | Part 3, case 3 | Part 2, case 13 | Part 2 case 12 | Part 2, case 14 | Part 2, case 11 | Part 2, case 24 | part 2, case 23 | Part 2, case 2 | Part 2, case 1 | Part 3, case 11 | Part 2, case 5 | Part 3, case 4 | Part 3, case 5 | Part 2, case 6 | Part 2, case 3 |
| **Center** | Berlin | Berlin | Berlin | Berlin | Berlin | Berlin | Freiburg | Düsseldorf | Berlin | Berlin | Berlin | Berlin | Vejle | Vejle | Heidelberg | Würzburg |
| **Age / years** | 54 | 54 | 77 | 20 | 30 | 57 | 43 | 30 | 20 | 47 | 48 | 34 | 33 | 43 | 56 | 54 |
| **Gender** | F | F | F | F | F | F | F | F | F | F | F | M | F | F | F | F |
| **Age at onset** | 50 | 28 | 66 | 6 | 28 | 53 | 42 | 30 | 17 | 40 | 44 | 22 | 30 | 34 | 47 | 48 |
| **Previous diagnosis** | rON | rON | rON | rON | CRION | rON | CRION | ON | NMOSD | NMOSD | NMOSD | NMOSD | NMOSD | MS/NMOSD | NMOSD | NMOSD |
| **AQP4-IgG** | - | - | - | - | - | - | - | - | - | - | - | - | - | - | - | - |
| **MOG-IgG [26]** | + | + | + | + | + | + | + | + | + | + | + | + | + | + | + | + |
| **Ophthalmologic comorbidities** | - | - | + **^b^** | - | - | + **^c^** | - | - | - | - | - | - | - | - | - | - |
| **EDSS** | 2.5 | 7.5 | 3.0 | 2.0 | 3.0 | 4.0 | 3.0 | 1.0 | 3.0 | 3.5 | 2.5 | 1.5 | 3.0 | 3.0 | 3.5 | 1.5 |
| **ON episodes** ^a)^ | 3 | 10 | 5 | 8 | 4 | 3 | 11 | 1 | 1 | 7 | 5 | 4 | 4 | 2 | 13 | 1 |
| **Myelitis episodes** ^a)^ | 0 | 0 | 0 | 0 | 0 | 0 | 0 | 0 | 2 | 2 | 1 | 1 | 5 | 10 | 1 | 2 |
| **Treatment escalation^d^** | - | + | + | + | + | + | + | - | + | + | + | + | + | + | + | + |
| **Ever contrast enhancement (MRI)? [25]** | + | - | n.d. | + | + | + | n.d. | n.d. | n.d. | + | n.d. | + | n.d. | n.d. | + | n.d. |
| **Ever optic nerve swelling (MRI or fundoscopy)? [25]** | + | + | n.d. | + | + | n.d. | + | + | n.d. | - | n.d. | + | n.d. | n.d. | + | n.d. |
| **Ever optic nerve atrophy (MRI or fundoscopy)? [25]** | n.d. | + | + | + | + | + | - | - | - | + | + | - | n.d. | n.d. | + | n.d. |
| **Worst VA ever during acute ON / logMAR [25]** | LP | NLP | NLP | 1.0 | 0.4 | CF | NLP | 0 | n.d. | NLP | LP | 0 | 0.7 | 1.0 | 1.0 | 1.0 |
| **VA worse eye at last follow-up or last known VA [25]** | blind | blind | blind | blind | blind | blind | blind | normal | n.d. | blind | blind | normal | severe | blind | blind | blind |

**Table S1 – Single patient overview**

a) Protracted relapses were registered as one episode.

b) Early stage dry macular degeneration in both eyes

c) Suspect for early stage glaucoma

d) Medication other than acute relapse therapy (immunotherapy)

Abbreviations: F: female, (r)ON: (recurrent optic neuritis). CRION: Chronic relapsing inflammatory optic neuropathy. NMOSD: neuromyelitis optica spectrum disorders. AQP4-IgG: Aquaporin-4 antibodies. MOG-IgG: Myelin-oligodendrocyte-glycoprotein antibodies. EDSS: Expanded disability status scale. MRI: magnetic resonance imaging. VA: Visual acuity. n.d.: not documented. (N)LP: (No) light perception. CF: Counting fingers.
