# Supplementary material for: MOG-IgG in NMO and related disorders: a multicenter study of 50 patients. Part 4: Afferent visual system damage after optic neuritis in MOG-IgG-seropositive versus AQP4-IgG-seropositive patients
Source: J Neuroinflammation. 2016 Nov 1;13:282. doi: 10.1186/s12974-016-0720-6 (PMC5088645; doi:10.1186/s12974-016-0720-6)
Supplement: Additional file 2: Table S2. — Visual evoked potentials, visual acuity, and OCT results. a) Protracted relapses were registered as one episode; b) Early stage dry macular degeneration in both eyes; c) Suspected early stage glaucoma; d) Medication other than acute relapse therapy (immunotherapy). Abbreviations: AQP4-IgG = aquaporin-4 antibodies; CRION = chronic relapsing inflammatory optic neuropathy; EDSS = expanded disability status scale; F = female; MOG-IgG = myelin oligodendrocyte glycoprotein antibodies; NMOSD = neuromyelitis optica spectrum disorders; (r)ON = (recurrent) optic neuritis. (DOC 59 kb) [file 12974_2016_720_MOESM2_ESM.doc]

| **Patient No.** | 1 | | 2 | | 3 | | 4 | | 5 | | 6 | | 7 | | 8 | | 9 | | 10 | | 11 | | 12 | | 13 | | 14 | | 15 | | 16 | |
| --- | --- | --- | --- | --- | --- | --- | --- | --- | --- | --- | --- | --- | --- | --- | --- | --- | --- | --- | --- | --- | --- | --- | --- | --- | --- | --- | --- | --- | --- | --- | --- | --- |
| **Eye** | **OD** | **OS** | **OD** | **OS** | **OD** | **OS** | **OD** | **OS** | **OD** | **OS** | **OD** | **OS** | **OD** | **OS** ^c)^ | **OD** | **OS** | **OD** | **OS** | **OD** | **OS** ^c)^ | **OD** | **OS** | **OD** | **OS** | **OD** | **OS** | **OD** | **OS** | **OD** | **OS** | **OD** | **OS** |
| **ON episodes** | 3 | 2 | 6 | 8 | 5 | 1 | 8 | 0 | 2 | 2 | 2 | 2 | 1 | 10 | 0 | 1 | 1 | 0 | 2 | 5 | 3 | 2 | 2 | 2 | 4 | 1 | 1 | 1 | 10 | 5 | 1 | 1 |
| **Time since last ON / months** | 4 | 35 | 36 | 36 | 96 | 123 | 12 | n/a | 18 | 18 | 10 | 10 | 3 | 0 | n/a | 8 | 7 | n/a | 13 | 0 | 2 | 2 | 9 | 125 | 3 | 37 | 84 | 25 | 24 | 13 | 71 | 71 |
| **VEP latency P100 / ms** | 138 | 134 | 140 | *n.e.* | *n.e.* | 107 | 107 | 107 | 124 | 108 | *n.e.* | *n.e.* | 112 | 170 | 104 | 112 | 103 | 98 | 109 | *n.e* | 138 | *175* | 101 | 101 | 147 | 114 | *n.d.* | *n.d.* | *n.e.* | *n.e.* | *103* | *105* |
| **Visual acuity / logmar** | 0 | 0 | 3.0 | 3.0 | 2.0 | 0.1 | 0.7 | 0.5 | 0.6 | 0.5 | 0.7 | 2.0 | 0 | 0.7 | -0.1 | -0.1 | -0.2 | -0.2 | 0 | 1 | -0.1 | 0.1 | -0.2 | -0.1 | 0 | -0.2 | -0.2 | 0 | 0.7 | 0.4 | 0 | 0.1 |
| **pRNFL thickness / µm** | 42 | 65 | 38 | 44 | 47 | 85 | 38 | 51 | 36 | 43 | 38 | 54 | 111 | 60 | 77 | 69 | 63 | 88 | 46 | 30 | 45 | 36 | 57 | 55 | 57 | 99 | 88 | 96 | 36 | 36 | 86 | 77 |
| **GCIP / mm³** | 1.39 | 1.71 | 1.21 | 1.22 | 1.26 | 1.64 | 1.27 | 1.4 | 1.19 | 1.24 | 1.16 | 1.05 | 1.95 | 1.67 | 1.57 | 1.42 | 1.49 | 1.76 | 1.23 | 1.08 | 1.31 | 1.03 | 1.69 | 1.70 | 1.38 | 2.12 | 1.82 | 2.03 | 1.12 | 1.18 | 1.99 | 1.89 |
| **INL / mm³** | 1.28 | 1.12 | 1.01 | 1.04 | 1.08 | 0.99 | 1.01 | 0.99 | 1.03 | 1.02 | 1.12 | 1.11 | 0.97 | 1.01 | 0.93 | 0.93 | 0.87 | 0.87 | 1.11 | 1.15 | 0.92 | 0.80 | 1.03 | 1.02 | 1.14 | 1.06 | 0.99 | 0.97 | 1.06 | 1.08 | 1.11 | 1.10 |
| **ORL / mm³** | 5.24 | 5.22 | 4.47 | 4.5 | 5 | 4.88 | 4.9 | 4.9 | 4.79 | 4.7 | 4.81 | 4.9 | 4.93 | 4.98 | 4.92 | 4.95 | 4.35 | 4.35 | 5.06 | 5.17 | 4.87 | 4.80 | 4.87 | 4.79 | 5.09 | 5.12 | 4.7 | 4.67 | 4.7 | 4.66 | 5.23 | 5,28 |
| **Macular microcysts** | + | - | - | + | - | - | - | - | - | + | - | - | - | - | - | - | - | - | + | + | - | - | - | - | + | - | - | - | - | - | - | - |

Table S2.
